# Artificial Respiration in Apoplexy

**Published:** 1875-08

**Authors:** 


### Artificial Respiration in Apoplexy.

In a recent number of the *Gazetta d' Italia*, Dr. Corso, the assistant to the chair of Physiology at Florence, advocates the use of artificial respiration in cases of "fulminant" apoplexy, and of compression of the brain by hemorrhage or other causes. The immediate cause of his remarks was a case related in the same paper, in which a Dr. Despalles, of Brussels, employed the inhalation of oxygen in a case of apoplexy with hemiplegia with a successful reresult, the sensibility and power of movement returning in four hours. Dr. Corso claims that artificial respiration was first employed for apoplexy in Prof. Schiff's laboratory, and quotes the opinion expressed by Schiff in his lectures on the nervous system, published in 1866, that in cases of fulminant apoplexy produced by paralysis of the medulla oblongatta, artificial respiration should be used, and never bloodletting. In 1871, Schiff also made numerous experiments on animals, and established the fact that the only way of preserving animals suffering from apoplexy, due to compression of the brain, from certain death, was artificial respiration. The explanation of this fact is that the immediate cause of death is paralysis of the respiratory centres by compression of the medulla oblongatta, and the consequent non-oxygenation of the blood. The advantage of artificial respiration over the inhalation of oxygen is that it lowers the pressure of the blood in the intra-cranial vessels, and thus lessens the tendency to further hemorrhage.

In support of these views Dr. Corso relates a case in which he claims to have employed artificial respiration for the first time in such cases in the human subject. It was that of a lady who fell backwards and sustained a fracture of the skull. When seen, she was cyanotic, insensible, breathing slowly and superficially, the pulse also was infrequent and very feeble, and death seemed imminent. Artificial respiration was immediately performed, and continued for twenty minutes; under its influence the heart's action increased in force and frequency, the respiration became natural, and consciousness returned. The patient did not, however, completely recover, but died fifteen days afterwards from the laceration of the brain caused by the hemorrhage and fracture. Dr. Corso points out that had it not been for the irreparable lesions produced by the injury, the patient would have lived, and the artificial respiration give the brain and the medulla oblongatta time to return to their normal functions, and hence a better chance of recovery, at any rate from the immediate danger.

There is no question that in many cases of coma due to compression of the brain, and also in that produced by opium or alcoholic poisoning, artificial respiration might be used with advantage; perhaps also in cases of so-called "congestive" apoplexy; but it seems to us open to grave doubt whether it would be applicable to cases of severe cerebral hemorrhage. The fear of increasing the hemorrhage should make us hesitate to adopt it until clearer proof is given that the restored action of the heart would not increase the pressure in the cerebral arteries and destroy that quiescence in which seems to lie the only chance of recovery in many cases. The difficulty in diagnosing the exact cause of the apoplexy will also stand in the way of any general employment of this method, except where it affords the only chance of obviating immediate death. *London Lancet.—Phil. Medical Times.*

